# Morphologic predictors of mandibular changes induced by Sander's Bite Jumping Appliance

**DOI:** 10.1111/ocr.12850

**Published:** 2024-08-23

**Authors:** Vincenzo D'Antò, Giorgio Oliva, Roberto Rongo, Rosaria Bucci, Stefano Martina, Lorenzo Franchi, Rosa Valletta

**Affiliations:** ^1^ Department of Neuroscience, Reproductive Sciences and Oral Sciences, Section of Orthodontics University of Naples Federico II Naples Italy; ^2^ Department of Medicine and Surgery and Dentistry University of Salerno Scuola Medica Salernitana Salerno Italy; ^3^ Department of Experimental and Clinical Medicine University of Florence Careggi Florence Italy

**Keywords:** Class II therapy, mandibular changes, prediction model, Sander's Bite Jumping Appliance

## Abstract

**Aim:**

This study aimed to identify pretreatment cephalometric variables as possible predictors of the mandibular length increase in Class II patients with mandibular retrusion, treated by means of the Bite Jumping Appliance (BJA).

**Materials and Methods:**

Forty‐three subjects (22 males and 21 females) with Class II malocclusion, treated with a BJA, were selected on the basis of the following inclusion criteria: full Class II molar relationship, Overjet (OVJ) ≥ 6 mm and a skeletal Class II malocclusion with mandibular retrusion at the start of the treatment (T0); cervical vertebral maturation stage 2 or 3 at time 0 (T0). The following mandibular structural features were measured on lateral cephalograms at time 0 and time 1 (15 months of treatment): the width and height of the mandibular symphysis and its width/height ratio, the width and height of the mandibular ramus and its width/height ratio, the antegonial notch depth and the Condilion–Gonion–Menton (Co–Go–Me) angle. Post‐treatment changes were assessed by Pancherz's cephalometric analysis, evaluating the increases in mandibular length. A regression statistical model was used to test the association between morphologic variables and mandibular length changes.

**Results:**

At T1, a significant increase in mandibular length (7.1 + 3.4 mm, *p* < .001) was measured. A significant negative association between the pretreatment Co–Go–Me angle and mandibular length change was found (*p* < .05). IMPA angle was negatively associated with mandibular length change. All the others morphological feature were not statistically related to mandibular length change.

**Conclusion:**

Co–Go–Me angle and IMPA angle at T0 can be used as predictors for mandibular response to the treatment with BJA.

## 1 INTRODUCTION

Approximately 80% of Caucasian patients with skeletal Class II malocclusion present mandibular retrusion.^1^, ^2^ As a consequence, the main goal of the treatment in these patients should be mandibular advancement.

A wide range of functional appliances aimed to stimulate mandibular growth by forward posturing of the mandible is available to correct Class II malocclusion.^3^ However, the efficacy of the functional orthopaedic treatment and the effects determined by the functional appliances are some of the most debated topics in the orthodontic literature, with the most controversial results.^4^ The effects can be classified as dentoalveolar and skeletal. Many studies have demonstrated the efficacy of orthopaedic treatment in inducing dentoalveolar compensations for the correction of malocclusion; the skeletal effects are more limited and still under study.^4^ The dento‐alveolar compensation affects both the lower and upper elements. The skeletal component is primarily expressed at the mandibular level. Although there is sufficient evidence that functional appliances, and in particular the twin block, decrease the OVJ, the clinical significance of the mandibular skeletal effects is still questioned, with large variability between studies.^3^, ^4^, ^5^ Predictive models in medicine are statistical tools designed to analyse patterns in data, allowing for predictions about future outcomes based on those patterns. These models can be of various types. Linear regression, which predicts a continuous response variable from one or more explanatory variables, is the most used prediction model for continuous type of data. The essence of a predictive model is its ability to process input data through statistical algorithms, generating predictions that can then guide clinical decisions. This makes them invaluable in medical research and practice for predicting patient outcomes, treatment responses, and disease progression, enhancing both diagnoses and prognoses.^6^ It has been observed that treatment success with functional appliances depends on a great number of variables: patient compliance for removable appliances,^7^ skeletal maturity,^3^, ^8^ and severity of the baseline conditions.^3^

Ahlgren found that poor cooperation was one of the main reasons for treatment failure.^9^ Bondevik attempted to identify the factors that influence the success of functional appliance treatment outcomes and found that good cooperation was the only variable associated with a satisfactory result.^10^ Regarding skeletal maturity, it has been proposed that a greater increase in mandibular growth occurs when the functional jaw orthopaedics treatment is performed during the pubertal growth spurt.^11^ Despite good compliance and adeguate treatment timing, some individuals with skeletal Class II malocclusion may present an unfavourable growth trend, as a consequence of a clockwise rotation growth pattern.^12^ Since an increased overbite may be an indicator of an inherent pattern of upward and forward growth rotation of the mandible, Caldwell and Cook investigated the relation between the pretreatment overbite (OVB) and the reduction of the overjet (OVJ) and showed that the overbite (OVB) is the variable most strongly related to the reduction of the overjet in growing patients treated with twin block appliance.^13^ Furthermore, it has been proposed that pretreatment overbite and the vertical height of the mandibular ramus were associated with a good prognosis for functional jaw orthopaedics outcomes.^14^ Franchi and Baccetti found that a Class II patient with a pretreatment Condilion–Gonion–Menton° (Co–Go–Me°) smaller than 125.5° is expected to be a “good responder” to functional jaw orthopaedics.^15^ This single predictor has been confirmed as effective in more recent sudies.^16^, ^17^ On the other hand, a retrospective study on 131 patients failed to find any relationship between mandibular morphology and favourable skeletal responses to twin block therapy.^18^ Furthermore, Ruf and Pancherz found no statistical differences for either dental or skeletal parameters between hypodivergent and hyperdivergent subjects treated with the Herbst appliance.^19^ Despite the contrasting results of the aforementioned studies, there is still a common belief in a good response to Class II treatment in forward growth rotation patients rather than in backward ones. Bjork drew attention to the possibility of predicting mandibular growth patterns by looking at some specific anatomic mandibular structures. Some of them (condylar head inclination, shape of the lower border of the mandible, inclination of the symphysis) are in some way anatomical feature related to forward vs backward mandible growth tendency.^20^ Aki et al. evaluated the morphology of mandibular symphysis as a predictor of the direction of mandibular growth.^21^ These authors reported that a mandible with an anterior growth direction was associated with a small height, large depth, small ratio, and large angle of the symphysis. Conversely, a posterior growth direction was associated by same authors^21^ to a large height, small depth, large ratio, and small angle of the symphysis. Singer et al. detected significant differences in mandibular growth between deep antegonial notch and shallow antegonial notch subjects.^22^ Kolodziej et al. found a statistically (but not clinically) significant negative relationship between antegonial notch depth and subsequent horizontal growth of the maxilla and mandible in untreated growing patients.^23^ A recent randomized clinical trial has shown that Sander's BJA is effective in determining a significant short‐term increase of mandibular growth in Class II individuals when compared with untreated controls.^24^ Even if BJA is an effective treatment for Class II treatment, the vast majority of Class II treatment prediction model were tested on twin‐block or Herbst appliances.^11^, ^13^, ^15^, ^16^ Sander's appliance^25^ might present some advantages over other functional devices, such as greater control of the occlusal plane and vertical dimension and It ensures an advanced position of the jaw even in mouth‐breathing patients.

The purpose of this study is to evaluate whether certain pretreatment mandibular structural characteristics, as measured on lateral cephalograms, can serve as predictors of successful mandibular growth in individuals treated with Sander's Bite Jumping Appliance (BJA).

## 2 MATERIALS AND METHODS

Forty‐three patients (22 males and 21 females), with an average age of 11.13 + 1.64, were included in the study. The study protocol was approved by the Ethics Committee of Naples Federico II (9619).

The patients were selected retrospectively on the basis of the following inclusion criteria: full Class II molar relationships, OVJ > 6 mm and skeletal Class II malocclusion with mandibular retrusion assessed by Fraenkel manoeuvre (aesthetic evaluation); cervical vertebral maturation stage (CVMS) 2 or 3; an age range of 11–14 years for boys and of 9–12 years for girls. The inclusion criteria differed between males and females to assess changes during the growth peak.^26^ The following conditions were considered as further exclusion criteria: periodontal diseases; orofacial inflammatory conditions; tooth agenesis; congenital syndromes and previous orthodontic treatment. These inclusion and exclusion criteria are similar to those used in a previous randomized controlled clinical trials conducted on this appliance.^24^

All the subjects were treated, by using a standard Sander's Bite Jumping Appliance with an acrylic cover of lower anterior teeth, at the Department of Neuroscience, Section of Orthodontics, of the University of Naples “Federico II” and at the Division of Dentistry of the Paediatric Hospital “Bambino Gesù” in Rome. The post‐treatment cephalograms were taken prior to commencement of fixed appliance therapy when a tendency to Class III molar relationship was achieved or after 15 months of treatment. The measurements were made blindly; the person who conducted the analyses was not involved in the treatment of the patients, did not perform the cephalometric analyses, and the data were anonymized before proceeding with the analyses.

The following mandibular structural features were measured on lateral cephalograms: the width (x) and height (y) of the mandibular symphysis and their ratio, the width (l) and height (h) of the mandibular ramus and their ratio, the antegonial notch depth, and the Co–Go–Me angle.

Symphysis height was calculated as follows (Figure S1): A line tangent to pogonion and perpendicular to Go‐Me was used as the long axis of the symphysis and a grid was formed with the lines of the grid parallel and perpendicular to the constructed tangent line. The superior limit of the symphysis was set at the lower incisor labial gingival border (cement‐enamel junction), the inferior at the most inferior point of the symphysis, the anterior at the most anterior point of the bony chin, and the posterior at the most inner point on the lingual border of the symphysis. The width of the symphysis was calculated as the distance from the anterior to the posterior limit on the grid.

Mandibular ramus width was calculated as the distance from R‐1 (the deepest point on the anterior border of the ramus, located halfway between the superior and the inferior curves) to R‐2 (located on the posterior border of the ramus, opposite R‐1). Mandibular ramus height was calculated as the distance from R‐3 (the deepest point of the sigmoid notch, halfway between the anterior and the posterior curves) to R‐4 (opposite R‐3 on the inferior border of the mandible; Figure S2).

Antegonial notch depth was calculated as the distance between the deepest part of notch concavity and a line passing through the two points of greatest convexity (ACP = anterior convex point; PCP = posterior convex point) on the inferior border of the mandible (Figure S3).

**FIGURE 1:**
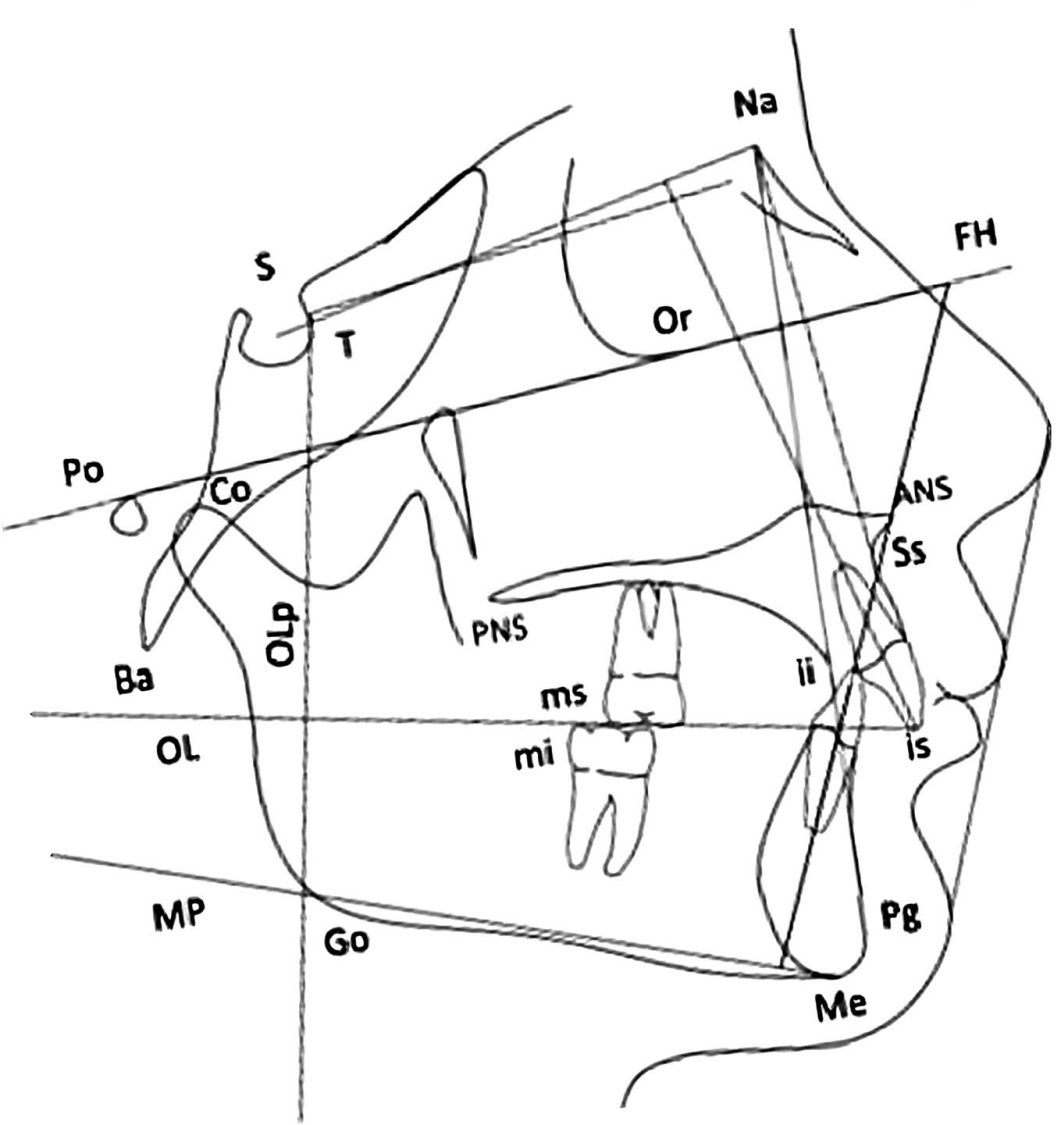
Cephalometric analysis. Landmarks: ANS (anterior nasal spine), the tip of the anterior nasal spine; Ba (basion), the midsagittal point of the anterior margin of the foramen magnum; Co (condyle), most superoposterior point on the curvature of the condylar head; where there was a double projection to two points, the midpoint was used; ii (incision inferius), incisal tip of the most prominent mandibular central incisor; is (incision superius), incisal tip of the most prominent maxillary central incisor; mi (molar inferius), distal contact point of the mandibular permanent first molar determined by a tangent perpendicular to the occlusal line (OL) – where there was a double projection to two points, the midpoint was used; ms (molar superius), distal contact point of the maxillary permanent first molar determined by a tangent perpendicular to OL – where there was a double projection to two points, the midpoint was used; Pg (pogonion), most anterior point on the bony chin determined by a tangent perpendicular to the OL; Ss (subspinale), deepest point on the anterior contour of the maxillary alveolar projection; Sella (S), center of the hypophyseal fossa; N (Nasion), most anterior point of the junction of the nasal and frontal bone (frontonasal suture); Or (Orbitale), lowest point of the inferior margin of the orbit; Po (Porion), most superior point on the anatomical external auditory meatus; Go (Gonion), midpoint of the curvature of the angle of the mandible; Me (Menton), most inferior point of the mandibular symphisis; PNS (posterior nasal spine): The tip of the posterior nasal spine; T (T point), most superior point of the anterior wall of the sella turcica at the junction with tuberculum sella. Reference lines: FH (Frankfurt horizontal), line connecting the Po point to the Or point; MP (mandibular plane), line connecting the Me point to the Go point; SN (sella nasion line), line through S and N; OL (occlusal line), line through the is point and the distobuccal cusp of the maxillary permanent first molar; OLp (occlusal line perpendicular), line perpendicular to the OL through the T; PP (palatal plane), line connecting ANS and PNS. Linear distances/skeletal landmarks: Ss/OLp, position of the maxillary base; Pg/OLp, position of the mandibular base; Co/OLp, position of the condylar head; Pg/OLp + Co/OLp, sagittal mandibular length. Linear distances/dental landmarks: is/OLp position of the maxillary central incisor; ii/OLp, position of the mandibular central incisor; ms/OLp, position of the maxillary permanent first molar; mi/OLp, position of the mandibular permanent first molar.

All of these measurements were collected by a single operator on digitized cephalograms using a digital calliper (Screen Calliper version 4.0). Cephalometric analysis was performed by the same operator, who had been extensively training in electronic cephalomethic analysis, using Dolphin Imaging 11.0 software (Chatsworth, CA, USA). Dentoalveolar, skeletal, and vertical changes with treatment were evaluated using the cephalometric landmarks and lines traced on pre‐treatment and post‐treatment radiographs superimposed following Pancherz's method (Figure 1).^27^

Variables for dental changes within the maxilla and within the mandible were calculated as follows: is/OLp minus Ss/OLp, change in position of the maxillary central incisor within the maxilla; Ii/OLp minus Pg/OLp, change in position of the mandibular central incisor within the mandible; Ms/OLp minus SS/OLp, change in position of the maxillary permanent first molar within the maxilla; Mi/OLp minus Pg/OLp, change in position of the mandibular permanent first molar within the mandible. For all of the linear measurements, the OL and the OLp of the initial radiograph were used as a reference grid. The grid was then transferred from the pretreatment radiograph to the post treatment one by superimposing on the nasion‐T point line, with the T point as the registering point. All of the measurements were made parallel to the OL. Differences in T1‐T0 linear measurements were recorded according to Pancherz's method.^27^

### 2.1 Statistical analysis

Mean, standard deviations, and minimum and maximum values were computed for all cephalometric parameters and morphological variables considered as predictors for mandibular growth in the current study. The method error for measurements of morphological features of the mandible was computed in 16 individuals using the Dahlberg Formula by collecting duplicate measurements at one‐week intervals. Error was on average 0.23 +/− 0.20 mm, and ranged from 0.007 mm for the width/height ratio of the mandibular symphysis to 0.58 mm for the measurement of the width of the mandibular ramus. A T‐test was used to assess cephalometric changes with treatment. Predictors for mandibular growth were: width, height of mandibular symphysis (SY‐width and SY‐ height); width, height of mandibular ramus (RM‐width and RM‐height), antegonial notch depth (AntNotch), Co–Go–Me°, IMPA, PP‐MP°. A high degree of correlation between these variables is suspected. To resolve this issue, a correlation matrix for the predictors will first be analysed, to exclude the most correlated ones. In addition to these predictors, gender, and age at time T0 will be added to the model as confounders. After the regression, a “stepforward” approach will be applied to select the most significant predictors. For the stepforward regression AIC (“Akaike Information Criterion”), a final model will be created based on these predictors. Although the dependent variable was a continuous variable, there was the option to discretize it and use a classification method, a linear regression model was ultimately used because there was no consensus among authors or in the literature on how to properly perform the discretization.

## 3 RESULTS

**FIGURE 2:**
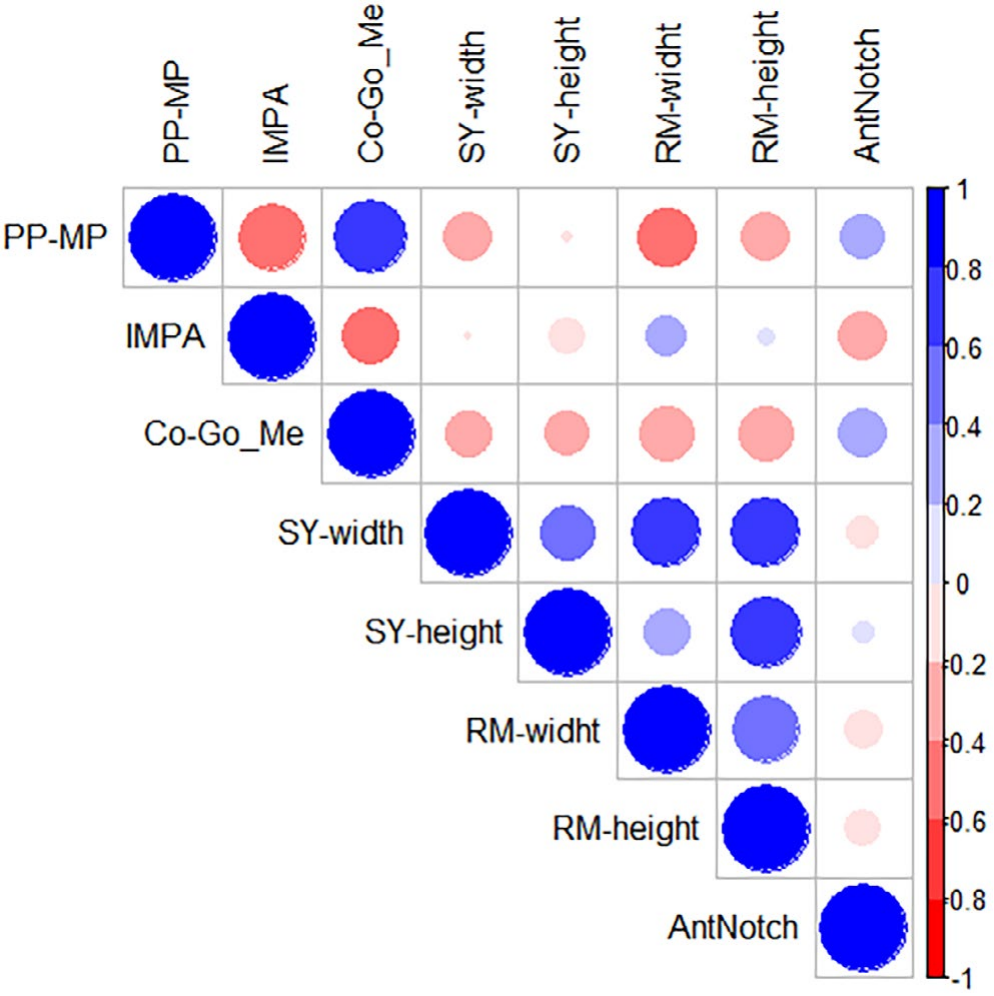
Correlation matrix of the predictors. Bigger points are referred to stronger correlation. Blue for positive correlation, Red for negative correlation. Number values at the right scale are referred to the Pearson correlation coefficient.

Skeletal and dental measurements at T0 and T1 and their changes with treatment are reported in Table 1. Mandibular length showed a significant increase during treatment (Pg/OLp + Co/Olp = 7.1 ± 3.4 mm, *p* < .001) as a result of increased Pg/OLp and increased Co/OLp. Vertical jaw relationships did not change significantly during treatment (MP‐FH° = 0.2 ± 2, *p* = .145; SN‐MP = −0.5 ± 2.6, *p* = .467). The appliance determined an improvement of sagittal dental relationships by producing a significant overjet reduction (−4.6 ± 2.6 mm, *p* < .001), a molar relation improvement (−4.9 ± 2.6, *p* < .001) with a small proclination of the lower incisors (4.8 ± 5.4, *p* < .001) and a retroclination of the maxillary incisors (−4.3 ± 6.2, *p* < .001). Figure 2 shows the correlation matrix between the analysed predictors (correlation coefficients are found in Table S1). Given the high correlation with other variables, the PP‐MP, SY‐height and RM‐width predictors were not used for the multiple regression model. Table 2 shows the results of the multiple linear regression. The only two predictors to be statistically significant are Co–Go–Me and IMPA. Table 3 shows the results of the stepforward predictors selection.

**TABLE 1:** Descriptive statistics for the variables examined. Linear measurements are in mm. T0 is the initial cephalometric analysis, T1 is related to the post‐treatment cephalogram.

Measurement	T0 (mean ± SD)	T1 (mean ± SD)	T1–T0 (mean ± SD) – 15 months	*p* Value
Overjet (is/OLp – ii/OLp)	7.9 ± 1.91	3.6 ± 1.5	−4.6 ± 2.6	<.001^a^
Molar relation (ms/OLp − mi/OLp)	2.2 ± 1.8	−2.6 ± 2.3	−4.9 ± 2.6	<.001^a^
Maxillary Base (Ss point to OLp)	67.5 ± 5.3	70.5 ± 5.2	2.9 ± 3.1	<.001^a^
Mandibular base (Pg/OLp)	67.3 ± 6.1	73.1 ± 7.2	5.9 ± 3.4	<.001^a^
Condylar head (Co/OLp)	13.4 ± 3.2	14.9 ± 3.8	1.2 ± 2.9	.019
Mandibular lenght (Pg/OLp + Co/OLp)	92.3 ± 12.8	100.5 ± 15.4	7.1 ± 3.4	<.001^a^
Mandibular length (Co − Pg)	97.8 ± 8.2	105.7 ± 8.8	7.9 ± 4.0	<.001^a^
Mandibular height (Co − Go)	50.6 ± 5.2	54.5 ± 6.4	3.8 ± 3.1	<.001^a^
Maxillary incisor (is/OLp − Ss/OLp)	8.5 ± 2.7	7.6 ± 2.9	−1.0 ± 2.0	<.001^a^
Mandibular incisor (ii/OLp − Pg/OLp)	0.3 ± 2.8	0.6 ± 3.6	0.3 ± 2.3	.310
Maxillary molar (ms/Olp − Ss/OLp)	−35.2 ± 9.6	−37.6 ± 10.2	−2.4 ± 2.8	<.001^a^
Mandibular molar (mi/OLp − Pg/OLp)	−37.2 ± 10.2	−37.7 ± 10.6	−0.4 ± 2.4	.800
SN‐MP (°)	30.0 ± 5.6	30.0 ± 5.8	0.2 ± 2.5	.467
MP‐FH (°)	22.6 ± 4.6	22.0 ± 4.7	−0.5 ± 2.6	.145
U1/SN (°)	108.6 ± 6.1	104.6 ± 6.6	−4.3 ± 6.2	<.001^a^
IMPA (°)	96.8 ± 7.04	101.3 ± 5.8	4.8 ± 5.4	<.001^a^
L1_FH (°)	60.7 ± 5.4	56.6 ± 5.7	−4.5 ± 5.3	<.001^a^
PP‐MP (°)	24.2 ± 4.7	23.8 ± 5.1	−0.3 ± 2.4	.859

*Note*: Significance level was set at *p* < .05.

^a^ Statistically significant.

**TABLE 2:** Multiple linear regression results.

Coefficients	Estimate	Std. Error	Pr(>|*t*|)
(Intercept)	94.11	22.75	.001
Age	−0.59	0.49	.23
Sex	0.78	0.23	.52
IMPA	−0.19	−0.09	.04^a^
Co–Go–Me	−0.47	−0.11	.001^a^
SY‐height	−0.11	0.24	.63
RM‐widht	−0.01	0.16	.90
AntNotch	−0.03	0.73	.95

^a^ Statistically significant.

**TABLE 3:** Stepforward results; deviance of the fit statistics and corresponding AIC for each step of the anlaysis.

Step	Deviance	AIC
		119.3674
+Co–Go–Me	170.2911	109.4007
+IMPA	47.34383	107.3634
+age	35.06234	106.1016

The regression model selected by the stepforward analysis includes three predictors: Co–Go–Me (which corresponds to the greatest reduction in the AIC parameter), IMPA and age.

Table 4 reports the regression model fitted with these three predictors only.

**TABLE 4:** Final multiple linear regression.

Coefficients	Estimate	Std. Error
(intercept)	88.07	18.44
Co–Go–Me	−0.45	0.09
IMPA	−0.18	0.08
Age	−0.65	0.37

The adjusted R‐squared for this model is 0.32.

## 4 DISCUSSION

The effectiveness of Class II therapy using mandibular advancement devices is influenced by several factors, including the age of the patient, their morphological characteristics, and the duration of time the device is worn.^8^, ^15^, ^28^ The present study investigated the morphological factors that influence the skeletal outcomes obtained using the BJA in growing patients. The average values of mandibular growth reported in this study are in line with those reported by Martina and colleagues.^24^ The referenced clinical trial found an average growth of 6.4 ± 2.3 mm in the treated group and 3.5 ± 2.5 mm in the untreated group. The growth observed in the present study, 7.1 ± 3.4 mm, aligns with the findings reported by the aforementioned researchers. Despite the agreement between the growth rate of our sample and the one reported in the literature, the lack of a control group is a limitation of this study. Among all the mandibular morphologic variables examined, the Co–Go–Me angle showed the most important correlation with mandibular changes (Pg/OLp + Co/OLp) in Class II subjects treated with BJA. This result is in agreement with a previous study by Franchi and Baccetti.^15^ The findings indicate that new Class II patients at CS3 with a pretreatment Co–Go–Me angle smaller than 125.5° are likely to respond positively to treatment involving functional jaw orthopaedics, which can promote supplementary mandibular growth. Conversely, new Class II patients at CS3 with a pretreatment Co–Go–Me angle greater than 125.5° are expected to exhibit a poor treatment response. Despite the same results, Franchi and Baccetti used a sample made of subjects treated with different kinds of functional orthopaedics appliances (twin block, stainless steel crown Herbst and acrylic splint Herbst), whereas our sample included only subjects treated by Sander's Bite Jumping Appliance. The Co–Go–Me angle expresses the inclination of the condyle in relation to the mandibular base and, probably, this variable is more intimately linked to a forward/backward rotation pattern of the mandible and could, therefore, have a great influence on therapy.^15^ Ruf and Pancherz, instead, focused on the correlation between the mandibular plane angle and Class II correction by means of the Herbst appliance.^19^ The authors analysed and compared the sagittal dental and skeletal effects contributing to Class II correction in subjects with small (ML/NSL < 26°) or large (ML/NLS > 39°) pretreatment mandibular plane angles in a treatment period of 7 months. No statistically significant differences for either dental or skeletal parameters was found between hypodivergent and hyperdivergent ones. Surprisingly, mandibular length (Pg/OLp) was, on average, advanced 1.1 mm more in the high angles than in the low angles even if the difference was not statistically significative. Our investigation was carried out considering the whole orthopaedic treatment phase (15 months) and the sample did not include severe hyperdivergent subjects who usually are not undergoing BJA in our clinical practice. Moreover, we focused on morphologic mandibular features instead of the mandibular plane angle because this angle expresses the position of the mandible in relation to other cranial structures and maybe should not play a significant role in predicting individual responsiveness to functional jaw orthopaedics. Mandibular position relative to the bispinal plane was not evaluated as a predictor as it was highly correlated with other predictors such as Co–Go–Me and IMPA. The other mandibular morphologic variables examined did not show any statistically correlation with mandibular length changes (Pg/OLp + Co/OLp). Aki et al. evaluated the morphology of mandibular symphysis as a predictor of the direction of mandibular growth in a cross‐sectional study.^21^ They considered B point as the upper limit of the symphysis instead of lower incisor labial gingival border. We preferred the latter to have the entire vertical dimension of mandibular symphysis even if it depends largely by the amount of lower incisors eruption. Moreover, they found a correlation between symphysis morphology and the direction of the mandibular growth, especially in male subjects. However, this assessment relied heavily on cross‐sectional adult data without any orthopaedic intervention. Our study, instead, investigated a sample of growing Class II malocclusion treated with functional jaw orthopaedics. No correlation was found between antegonial notch depth and mandibular growth. Conversely, Singer et al.^22^ found a strong correlation between them. He based the results of his study on Bjork's implant studies^20^ reporting that mandibles with a forward growth tendency exhibit a surface of apposition below the symphysis and surface of resorption under the mandible angle. The opposite pattern occurred in the subjects with backward mandibular growth tendencies leading to concavity on the lower border of the mandible known as the antegonial notch. However, the sample used by Singer consisted of 50 growing subjects with extreme morphologic patterns (notch depths >3.0 mm or <1 mm), whereas the sample of our investigation was a non‐extreme population (notch depth mean: 1.2 ± 0.9). Kolodziej et al.^23^ found a statistically (but not clinically) significant correlation (0.40 < *r* < 0.47; *p* < .05) between antegonial notch depth and horizontal growth of the maxilla and mandible from adolescence to adulthood in an untreated group of adolescents without the bias to extremeness, but, due to the lack of clinical significance, the authors did not suggest the use of antegonial notch depth as a predictor of mandibular growth. The lack of clinical significance is due to the low variability of the predictor, which, combined with a low correlation coefficient, does not allow the use of the predictor except for extreme cases. Another potential drawback of using the anthegonial notch is the possibility of measurement errors due to changes in the mandible's spatial position between cephalograms. Since this variable was excluded from the regression model, potential errors will not be further investigated. In our study, a correlation was found between IMPA and mandibular growth. This correlation was not identified by Lombardo and coauthors.^16^ The role of the IMPA in predicting the capacity for mandibular growth stimulated by the appliance can be explained in various ways, particularly an increase in the inclination of the incisors prevents planning the mandibular advancement necessary for the complete correction of the skeletal defect. Although the device used in our study provides a different control of the incisal position (capping),^25^ this difference should be further analysed and the role of incisal proclination studied with further investigations. In this study, the best predictive model explains approximately 32% of the variability; other variables like compliance in appliance wear should be studied in future studies.^28^ This study may suffer of some limitations. Given the retrospective nature, it may overstate the likely effectiveness of the appliance. The retrospective nature of the study could lead to selection bias. To limit this, the same inclusion criteria of a previous RCT were used, but the study design still entails this limitation. Consequently, the findings obtained may be considered a best‐case scenario. The validity of the results is only related to Sander II appliance in a Caucasian population from South of Italy. Despite this, the Co–Go–Me value has been found to be significant for different orthodontic appliances by different groups of authors; indicating how this measure can be considered a useful aid for Class II treatment planning.

## 5 CONCLUSIONS

- The findings indicate an inverse relationship between the Co–Go–Me angle and mandibular growth changes in response to treatment. Class II patients with a smaller pretreatment Co–Go–Me angle are likely to exhibit a favourable response to Sander's Bite Jumping Appliance treatment, which can promote supplementary mandibular growth. Conversely, Class II patients with a larger pretreatment Co–Go–Me angle are expected to demonstrate a poor treatment outcome.
- IMPA showed a negative correlation with mandibular growth changes, this predictor should be further investigated.
- A three predictors model composed by Co–Go–Me°, IMPA and age was the most effective multiple linear model (adjusted *R* ^2^: 0.32): “Mandibular length changes = 88.07 −0.45 (Co–Go‐Me) – 0.18 (IMPA) −0.65 (age)”.
- Mandibular symphysis, Mandibular ramus, and antegonial notch depth did not show any correlation with mandibular growth changes and they should not be used as growth predictors.

## AUTHOR CONTRIBUTIONS

VD carried out the experimental studies. RR and RB wrote a draft of the manuscript and carried out the literature review and revised the draft text. GO carried out the statistical analysis and prepare results section. RV carried out the general study planning and prepared the article for publication. LF reviewed all work, edited and prepared the article for publication. All authors read and approved the final version of the manuscript.

## FUNDING INFORMATION

This research received no external funding.

## CONFLICT OF INTEREST STATEMENT

The authors have no conflicts of interest to declare. All co‐authors have seen and agree with the contents of the manuscript and there is no financial interest to report.

## ETHICAL APPROVAL

The study protocol was approved by the Ethics Committee of the University of Naples Federico II (9619).

## Supporting information


Figure S1.

Figure S2.

Figure S3.

Table S1.

## Data Availability

The data that support the findings of this study are not publicly available due to privacy and ethical restrictions.
