# Supplementary figures and images for: Morphologic predictors of mandibular changes induced by Sander's Bite Jumping Appliance
Source: Orthod Craniofac Res. 2024 Aug 23;28(1):67–74. doi: 10.1111/ocr.12850 (PMC11701945; doi:10.1111/ocr.12850)

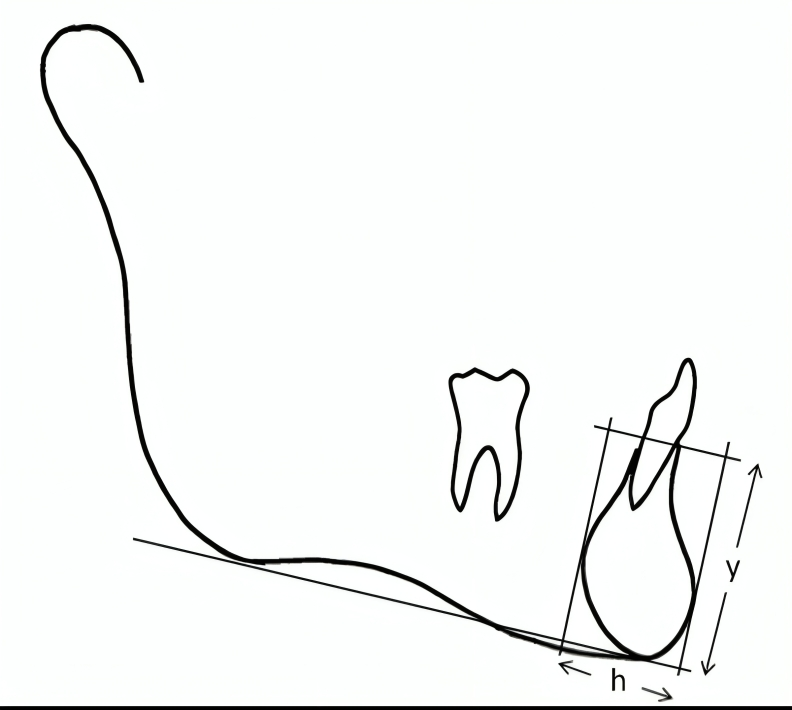

Supplement: Supplementary file 1 — Figure S1. [file OCR-28-67-s004.tif]

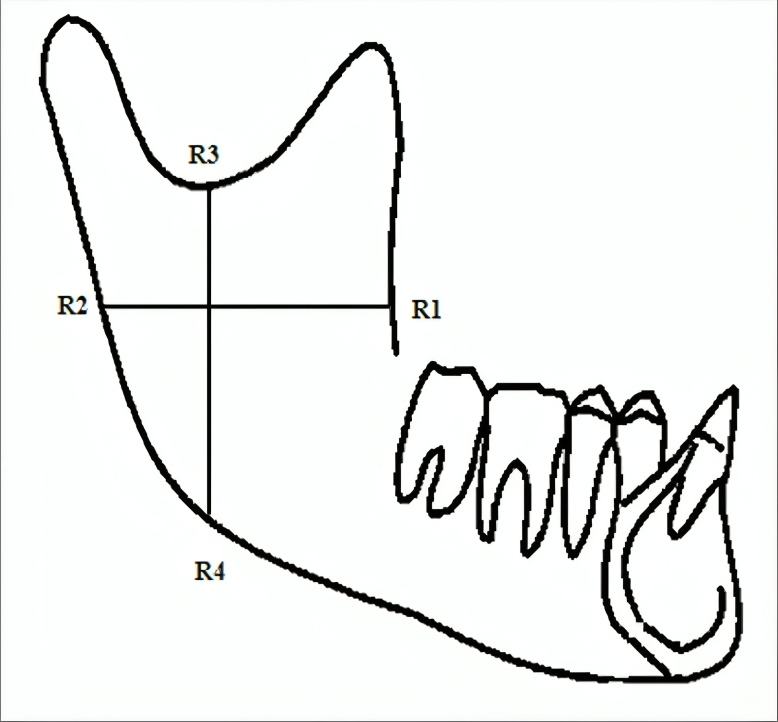

Supplement: Supplementary file 2 — Figure S2. [file OCR-28-67-s003.tif]

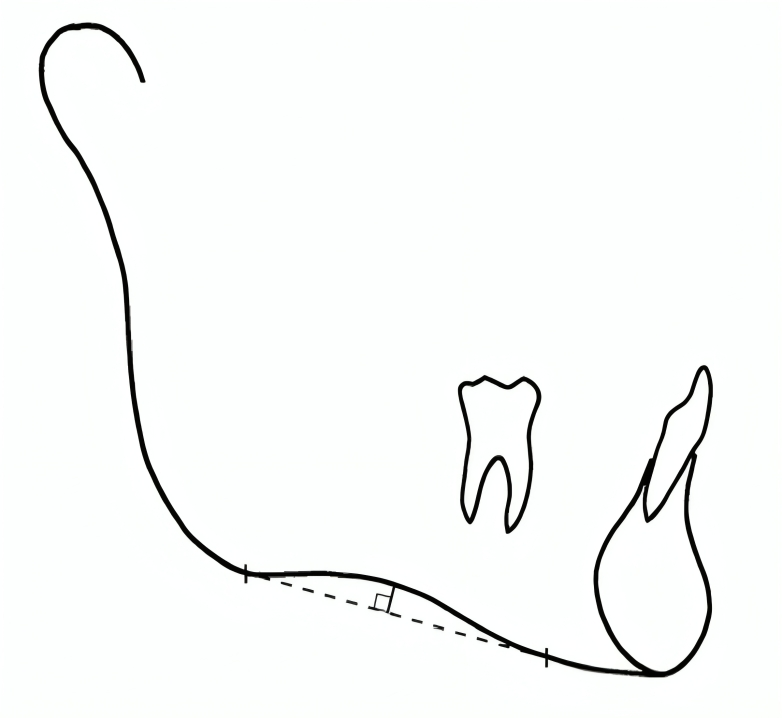

Supplement: Supplementary file 3 — Figure S3. [file OCR-28-67-s002.tif]
