# Supplementary material for: Morphologic predictors of mandibular changes induced by Sander's Bite Jumping Appliance
Source: Orthod Craniofac Res. 2024 Aug 23;28(1):67–74. doi: 10.1111/ocr.12850 (PMC11701945; doi:10.1111/ocr.12850)
Supplement: Supplementary file 4 — Table S1. [file OCR-28-67-s001.docx]

| **Correlation** | **PP-MP** | **IMPA** | **Co-Go_Me** | **SY-width** | **SY-height** | **RM-widht** | **RM-height** | **AntNotch** |
| --- | --- | --- | --- | --- | --- | --- | --- | --- |
| PP-MP | 1 |  |  |  |  |  |  |  |
| IMPA | -0,573098928 | 1 |  |  |  |  |  |  |
| Co-Go_Me | 0,691853923 | -0,431561083 | 1 |  |  |  |  |  |
| SY-width | -0,311646107 | -0,008103596 | -0,27733681 | 1 |  |  |  |  |
| SY-height | -0,017456831 | -0,182124864 | -0,268361631 | 0,40476782 | 1 |  |  |  |
| RM-widht | -0,454447904 | 0,223395863 | -0,389590514 | 0,626402388 | 0,283794892 | 1 |  |  |
| RM-height | -0,300800191 | 0,044328696 | -0,385714004 | 0,628368246 | 0,677633406 | 0,576205082 | 1 |  |
| AntNotch | 0,261000611 | -0,304667406 | 0,323103394 | -0,139183919 | 0,061627169 | -0,194395003 | -0,171830378 | 1 |
